# Kinetic and Kinematic Differences in a Golf Swing in One and Both Lower Limb Amputees

**DOI:** 10.1515/hukin-2015-0089

**Published:** 2015-01-12

**Authors:** Petr Stastny, Adam Maszczyk, Kristina Tománková, Petr Kubový, Michaela Richtrová, Jakub Otáhal, Rostislav Čichoň, Aleksandra Mostowik, Piotr Żmijewski, Paweł Cięszczyk

**Affiliations:** 1 Palacky University in Olomouc, Faculty of Physical Culture, Tr. Miru 115, post. 771 11 Olomouc, Czech Republic; 2 Department of Theory and Practice of Sport; The Jerzy Kukuczka Academy of Physical Education in Katowice; Poland; 3 Charles University in Prague, Faculty of Physical Education and Department of Anatomy and Biomechanics, Laboratory of Extreme Loading; 4 Department of Physiology, Institute of Sport, Warsaw Poland; 5 Faculty of Physical Education and Health Promotion, University of Szczecin, Poland

**Keywords:** golf swing, lower limb amputee, handicap, X factor, S factor, O factor, disability

## Abstract

Amputee golfers need to cope with the absence of sole proprioception, a decreased range of swing motion and other factors which should be recognized for training purposes. The aim of this study was to determine the kinetic and kinematic differences in the golf swing in one leg and two legs amputees. The participants consisted of two males and one female at a professional or amateur level with a different degree of disability. Each participant was taped by 3D markers and performed five golf swings with the iron 6. The intraclass correlation coefficient (ICC) did not vary between individuals in kinematics, however, it was low in kinetic variables of two leg amputees. The Kendal rank correlation showed a significant relationship between the level of amputation and a large number of kinetic and kinematic variables such as X factor, O factor, S factor and individual body angles. The fluency and similarity of the golf swing did not depend on the level of amputation. One lower limb amputation did not seem to increase movement variability contrary to two lower limb amputation. The most variable parameter was a weight-shift in all golfers. The takeaway and horizontal force angle depended on the level of amputation rather than individual technique, thus, their modification by training may be difficult. Estimation of golf swing „mistakes” in amputees in respect to the leading arm in an early follow or late follow position appeared to be useless.

## Introduction

Sport performance in subjects with disabilities is influenced by the range of deteriorated movements and body functions, which these athletes have to resolve. Amputee golfers need to cope with the absence of sole proprioception, which is crucial for CNS quasistatic and dynamic equilibrium of the human body (Kovacikova et al., 2015) as a presumption of an accurate golf drive. Additionally, the location of amputation influences the range of swing motion and other factors which should be recognized for the purpose of training progress. Lower limb amputees may feel enormous discomfort due to lack of the information about the distribution of pressure between the foot and boot and change in weight distribution (weight-shift) (Gerych et al., 2013; Jelen et al., 2005).

The most significant difference may be found in one and two leg amputees, yet one leg amputees have at least a one proprioceptive support from the sole of the foot what allows better control of the centre of gravity than in two leg amputees. On the other hand, both leg amputees require similar proprioception from remaining lower limbs, while the stance condition might have a more consistent initial position. Movement pattern differences may also exist between below and above knee amputees for the presence or absence of knee joint proprioception.

Previous studies have indicated the key body positions (Neal and Wilson, 1985; Nesbit, 2005; Williams and Cavanagh, 1983) and electromyography characteristics (McHardy and Pollard, 2005) such as main joint angles, a force impulse during the ball hit and upper torso–pelvis separation called X-factor (Cheetham et al., 2001; Chu et al., 2010). X factor is the relative position of the biacromial line and both spina iliaca anterior superior lines in the horizontal plane, which should cross each other up to 50° or even more in the top swing position (Chu et al., 2010). According to our opinion, these factors are significantly influenced by lower limb disabilities, yet without exact knowledge to what extent.

The aim of this study was to determine the kinetic and kinematic differences in the golf swing in one leg and two legs amputee golfers. We estimated variability of the golf swing course and determined which movement parameter depended on the level of amputation. The findings of the present study may be useful in order to indicate which golf swing variable is determined by the level of athlete’s amputation and which is influenced by individual technique.

## Material and Methods

### Experimental Approach to the Problem

A four case study in a cross-sectional design was performed in a laboratory environment with each participant of a different degree of disability.

### Participants

The study sample consisted of 3 amputee golfers (two men and one women) at a professional or amateur level with a different degree of disability described in Table 1. The fourth subject was a healthy professional golf player used as a reference case. Informed written consent was provided by each participant and the testing protocol was approved by the local Committee of Ethics in accordance with the ethical standards of the Declaration of Helsinki (1983).

### Procedure

The warm-up procedure included individual trials of the golf swing for 10 min and dynamic stretching with trunk rotations. After the warm up, each participant was taped by 3D markers and performed five golf swings with the iron 6. A 60 s interval was allowed between trials with a renewed initial position. The swings were performed in a laboratory environment on an artificial turf tee box, as there are no significant biomechanic differences between the practice and competition trials besides the golf swing speed (Croix and Nute, 2008). Kinematic variables were measured by means of the six camera 3D passive markers system Qualisys (Qualisys AB, Gothenburg, Sweden) and kinetic variables were evaluated with the use of two force plates Kistler (Kistler Group, Winterthur, Switzerland). The participants stood on a separate force plate with each leg.

### Measures

**Figure 1:**
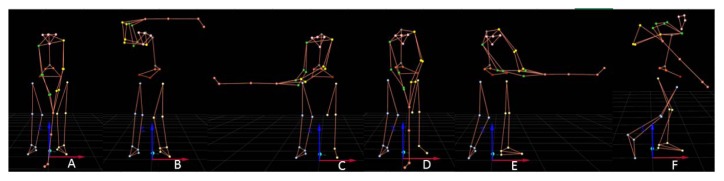
Selected movement position for kinematic analyses A = initial position, B = takeaway at the end of the back (top) swing, C = forward swing - the club in a horizontal position after the back swing, C = acceleration – the tee contact, D = early follow – a horizontal club position after the tee contact, E = late follow – the end of motion

The kinematic data were recorded at a frequency of 200 Hz using a six-camera Qualisys Oqus 3+ (Qualisys AB, Gothenburg, Sweden) infra-red motion analysis system and by a high speed digital camera. The cameras were spaced around the “tee” with two force plates in the middle. The force plates were connected to the Qualisys Track Manager interface software. Furthermore, the force acting on each leg was recorded using separated Kistler detectors with 1000 Hz recording frequency. Reflective markers measuring 19 mm in diameter were attached bilaterally on the subject’s skin overlying the following landmarks: the anterior superior iliac crest, the posterior superior iliac crest, the lateral knee, the medial knee, the lateral femoral epicondyles, the lateral malleolus, heels, the metatarsal head of the second toe, processus spinosus of L5, procesus spinosus C7, the acromion, the chin, the oss temporalis, the glabela, the lateral epicondyle of humerus, the medial epicondyle of humerus, the caput ulnae and procesus styloideus radii. Other markers were attached to the golf club at the top of the stick, in the middle of the stick, the heel of the club and the end of the club head. For further analysis, the virtual markers were placed in the middle of the crossline between the anterior superior and posterior superior iliac crest, the half distance between the superior posterior iliac crest and half distance between acromions. 3D kinematics were used to detect swing phases according to previous studies (Bechler et al., 1995; McHardy and Pollard, 2005), where the takeaway covered the movement from the initial position to the end of the back swing, the forward swing covered the movement from the top of the back swing to the club in a horizontal position, acceleration covered the movement from the horizontal club position to the tee contact, an early follow phase from the tee contact to the horizontal club and the late follow phase from the horizontal club after the tee contact to the end of motion. The kinematic data were collected in the following body positions (Figure 1):

A, initial position
B, takeaway - at the end of the back (top) swing
B, forward swing – the club in a horizontal position after the back swing
C, acceleration – the tee contact
D, early follow – a horizontal club position after the tee contact
E, late follow – the end of motion

Recorded variables were the main joint angles such as knee flexion/extension, hip flexion/extension, arm abduction/adduction, arm flexion/extension, elbow flexion/extension, impact speed and complex swing factors such as X factor, O factor and S factor. O factor was calculated as the angle between a 3-D line defined by the right and left anterior superior iliac spines and the horizontal plane. S factor was calculated as the angle between a 3-D line defined by the right and left acromion processes and the horizontal plane. The kinetic variables were recorded separately for each lower limb and they were expressed as the angle between a horizontal force vector and a horizontal plane for the right leg (Rx), the angle between a horizontal force vector and a horizontal plane for the left leg (Lx), the angle between a horizontal force vector and a sagittal plane for the right leg (Ry) and the angle between a horizontal force vector and a sagittal plane for the left leg (Ly). The weight shift during the golf swing was estimated by the vertical force ratio between right and left legs expressed in percentage.

### Statistical Analyses

All statistical analyses were performed using STATISTICA version 12 (StatSoft, Inc., Tulsa, OK, USA) with α = 0.05. Within subject reliability was estimated by mean and individual intraclass correlation coefficients (ICC_m_, ICC_i_) across 5 repetitions through all swing positions of each participant. These correlations were used to determine if the golf swing was stable within each subject (Table 2). Kendal rank-order correlations (Kendal tau b “Ƭ”) were used to determine the dependence of the movement ICCs, kinematics and kinetics during the golf swing on the amputation level. For this test, the level of amputation was regarded as one group of variables (predictors) numbered as follows: 1 for no-amputation, 2 for below knee amputation, 3 for femur amputation and 4 for both femur amputation. The Kendal’s Ƭ was used as this coefficient does not require any assumption of correlation linearity and is not dependent on the number of studied cases (Sheskin, 2003).

## Results

Both ICCs did not vary between participants 1, 2 and 3. The subjects had their specific positions with lower ICCs (higher movement variability) ( Table 2). Furthermore, these values did not show any relationship between the level of amputation and movement variability by ICC_m_ and ICC_i_. Participant 4 presented moderate or low ICC values for kinetic variables, what indicates insufficient movement stability (Table 2). The weight-shift was found to be the most variable parameter with moderate or low ICC values (Table 2).

The Kendal rank correlation showed a significant relationship between the level of amputation and X factor in the takeaway position (Ƭ = −0.88, p < 0.01), O factor in the takeaway position (Ƭ = −0.90, p < 0.01), O factor in the early follow position (Ƭ = 0.65, p < 0.01), O factor in the late follow position (Ƭ = 0.62, p < 0.01), S factor in the initial position (Ƭ = 0.54, p = 0.02) and S factor in the takeaway position (Ƭ = −0.66, p < 0.01). This relationship in complex swing parameters was accompanied by significant differences between the level of amputation and initial right knee flexion (Ƭ = 0.76, p < 0.01), right knee flexion in the takeaway (Ƭ = 0.56, p = 0.01), right knee flexion in the acceleration position (Ƭ = 0.61, p < 0.01), left knee flexion in the forward swing position (Ƭ = 0.62, p < 0.01), left knee flexion in the acceleration position (Ƭ = 0.66, p < 0.01), left knee flexion in the early follow position (Ƭ = 0.79, p < 0.01), left elbow flexion in the late follow position (Ƭ = 0.56, p = 0.01), left elbow flexion in the takeaway position (Ƭ = −0.56, p = 0.01), left elbow flexion in the forward swing position (Ƭ = −0.67, p < 0.01), right arm abduction in the acceleration phase (Ƭ = 0.59, p < 0.01), left arm abduction in the initial position (Ƭ = 0.59, p < 0.01), left arm abduction in the forward swing position (Ƭ = 0.77, p < 0.01), left arm abduction in the acceleration phase (Ƭ = 0.66, p < 0.01), left hip abduction in the acceleration phase (Ƭ = −0.53, p = 0.02), right hip abduction in the acceleration phase (Ƭ = 0.54, p = 0.02), right hip abduction during the early follow phase (Ƭ = 0.73, p < 0.01), right hip abduction during the late follow phase (Ƭ = 0.63, p < 0.01), right hip flexion in the acceleration phase (Ƭ = −0.90, p < 0.01), right hip flexion in the early follow phase (Ƭ = −0.62, p < 0.01), right hip flexion in the late follow phase (Ƭ = −0.53, p < 0.01), left arm flexion in the forward swing (Ƭ = −0.61, p < 0.01) and left arm flexion in the early follow phase (Ƭ = 0.60, p < 0.01). Kinetic variables showed significant differences between the level of amputation and the vertical force ratio in the late follow position (Ƭ = 0.50, p = 0.02), Rx angle in the forward swing position (Ƭ = 0.61, p < 0.01), Lx in the early follow position (Ƭ = 0.78, p < 0.01), Lx in the acceleration phase (Ƭ = 0.62, p < 0.01), Lx in the late follow position (Ƭ = 0.55, p = 0.01), Ry in the initial position (Ƭ = −0.55, p = 0.01) and Ly in the early follow position (Ƭ = −0.65, p < 0.01).

Other relationships were found between the acceleration speed and X factor at the acceleration position (Ƭ = 0.77, p < 0.01). The relationships between the level of amputation and movement kinetic and kinematic parameters are listed in Table 3.

## Discussion

The finding that the ICC did not vary between the golfers with a different level of amputation pointed to the sufficient level of individual technique in one leg amputees, but insufficient in both leg amputees for kinetics (Table 2). The fluency and similarity of the golf swing have been considered as ones of the key factors in golf performance (Meister et al., 2011), yet, they appeared independent of the level of amputation in the present study. If golfers have low swing variability (fluent swing), their rotatory parameters such as X, O and S factors should correlate with clubhead speed during acceleration (Meister et al., 2011; Myers et al., 2008). This dependence was found in our study (Table 3), however, not in the study by Kwon et al. (2013).

All complex factors were dependent on the level of amputation in the takeaway position, where the participants with both femur amputation showed lowest values (Table 3). This effect derived from the mechanical reduction of lower limb range of motion by the prosthesis. With regard to training application, the takeaway position seems to be determined, therefore its susceptibility to training is relatively low. This is a significant difference compared to golfers without disability, where the takeaway position is often trained prior to other skills. Other factors dependant on the level of amputation were the horizontal force angle, left arm (leading arm) flexion and left arm abduction (Table 3). There were also lower limb movement variables, which depended on the level of amputation, but this effect was expected due to the rigidity of used prosthetics. The horizontal force angle was dependant during the forward swing, acceleration and the early follow position, which means that the level of amputation was a determinant for this force. Thus, this part of movement should not be altered in training. The left arm abduction, left arm flexion and left elbow flexion were dependant in the acceleration position, the early follow and late follow position, respectively. This finding is important for actual training, as golfers are used to deduct the individual technique mistakes from early follow, late follow and other positions of the leading arm. This kind of deduction was found to be useless in amputee golfers. Previous simulations reported that delayed release of the club and leading arm could increase the club head velocity (Sprigings and Mackenzie, 2002), which is probably not applicable in lower limb amputees.

This weight-shift was recognized by the right and left foot vertical force ratio throughout the swing, which was influenced by the level of amputation only in the late follow position for force ratios. Thus, a decreased possibility of proprioception and movement coordination in amputees is evident after a drive rather than a takeaway, a swing and a clubhead impact.

This might be explained by athletes practice, as they referred the weight-shift as the key element in their training. On the other hand, importance of the weight-shift and need for its frequent training is one of the golf basics (Chu et al., 2010). Low-handicap golfers should be recognized by a larger (Kawashima et al., 1999; Koenig et al., 1994) and quicker (Okuda et al., 2002) weight-shift in the backswing, however, this issue was not addressed in the present study. The only finding was that the weight-shift was the most variable parameter considering that both leg amputee golfers showed a low level of force ratio change (weight shift) during the golf swing.

X factor should delay release of the arms and wrists towards the trunk forward movement, lateral tilting and weight-shifting during the swing (Chu et al., 2010), which should increase the speed of the clubhead in the acceleration phase. The subjects of the present study showed dependence of X factor on the level of amputation in the takeaway, yet, in any case X factor of one leg amputees was appropriate to their golf handicap when compared to the results of previous studies (Meister et al., 2011; Myers et al., 2008) performed on athletes without disability. Thus, X factor was found to be higher in professionals (Cheetham et al., 2001), low-handicap golfers (Watanabe et al., 1998), golfers with a high ball and swing velocity (Myers et al., 2008; Zheng et al., 2008), and professionals with a high driving distance.

Discussing study limitations we should indicate a small sample size considering that it did not cover all possible variations of amputation. Furthermore, the study design included only athletes using their own prosthetic equipment which they used on a daily basis. The movement variability might be much different if there was a comparison to the swing condition without prosthetics or with specially adopted ones. This study also did not estimate the differences between a “regular” and a hybrid golf swing, which might be useful for golf swing training (McHardy et al., 2006).

## Conclusion

The fluency and similarity of golf swing kinematics are not dependant on the level of amputation. One lower limb amputation did not increase movement variability in kinetics contrary to two lower limb amputation. The most variable parameter is the weight-shift in all golfers. The takeaway and the horizontal force angle were dependent on the level of amputation rather than individual technique, thus, their modification by training is difficult. Estimation of golf swing „mistakes” in amputees by the leading arm position in the early follow or late follow phase seems to be useless.

## Figures and Tables

**Table 1:** Participants’ characteristics

	Case 1	Case 2	Case 3	Case 4
Gender	male	male	male	female
Age (years)	21	41	45	44
Body mass (kg)	80	73	89	70
Body height (cm)	178	172	187	180
Dominant limb*	right	left	left	right
Side of the golf club	right	right	right	right
Amputation age (years)	non	8	30	4
Amputation degree	non	Right shin	Right femur	Both femur
Prosthesis type	non	Trias foot	C-leg + Trias foot	C-leg + Trias foot
Golf before amputation	---	no	no	yes
HCP	5.1	21.2	25	25

* regarding a one side amputee, the non-amputated limb is considered as dominant.

HCP = golf handicap.

**Table 2:** Within subject reliability of selected variables between individual golf swing trials

variable	Participant 1			Participant 2			Participant 3			Participant 4		
	ICC_i_	ICC_m_	SEM	ICC_i_	ICC_m_	SEM	ICC_i_	ICC_m_	SEM	ICC_i_	ICC_m_	SEM
X factor	0.93	0.97	2.91	0.54	0.78	3.11	0.96	0.98	4.25	0.58	0.80	2.42
O factor	0.97	0.99	1.94	0.95	0.98	1.19	0.68	0.86	1.12	0.72	0.88	0.31
S factor	0.98	0.99	6.32	0.91	0.97	5.41	0.90	0.96	6.91	0.86	0.95	1.54
Knee flex R	0.76	0.91	0.87	0.96	0.98	3.18	0.90	0.94	2.34	0.74	0.85	0.38
Knee flex L	0.71	0.78	0.88	0.8	0.8	1.99	0.98	0.99	2.12	0.74	0.78	0.39
Elbow flex R	0.97	0.99	8.79	0.99	0.99	8.61	0.98	0.99	9.41	0.97	0.99	9.51
Elbow flex L	0.99	0.99	9.81	0.96	0.99	7.13	0.99	0.99	10.21	0.99	0.99	7.72
Arm abd R	0.83	0.93	4.92	0.86	0.94	3.31	0.91	0.96	4.43	0.96	0.99	3.83
Arm abd L	0.99	0.99	7.53	0.94	0.97	5.92	0.98	0.99	7.51	0.82	0.93	4.41
Arm flexR	0.86	0.94	4.82	0.87	0.96	3.71	0.93	0.94	4.39	0.92	0.99	3.48
Arm flexL	0.91	0.97	7.81	0.95	0.98	6.14	0.94	0.98	8.21	0.93	0.99	6.72
Hip flex R	0.77	0.84	2.73	0.87	0.90	1.91	0.98	0.99	2.08	0.74	0.78	0.32
Hip flex L	0.79	0.82	2.25	0.88	0.89	1.93	0.96	0.98	2.14	0.74	0.78	0.34
Hip abd R	0.81	0.90	1.91	0.87	0.91	1.21	0.91	0.93	2.14	0.94	0.96	0.25
Hip abd L	0.78	0.85	2.15	0.89	0.92	1.19	0.89	0.95	2.15	0.95	0.99	0.19
Force ratio	0.48	0.73	0.14	0.92	0.97	0.18	0.37	0.63	0.39	0.12	0.30	0.28
Rx angle	0.58	0.81	3.44	0.71	0.92	4.52	0.75	0.90	3.22	0.34	0.61	4.7
Lx angle	0.94	0.98	1.13	0.47	0.73	1.12	0.5	0.13	0.98	0.1	0.42	0.25
Ry angle	0.66	0.85	1.47	0.75	0.90	1.02	0.56	0.79	1.89	0.51	0.76	0.27
Ly angle	0.63	0.83	0.51	0.98	0.99	0.52	0.99	0.99	0.62	0.37	0.41	0.38

ICCm =Mean intraclass correlation coefficient, ICCi = individual intraclass correlation coefficient, SEM = standard error of measurement, Rx = angle between the horizontal force vector and the horizontal plane for the right leg, Lx = angle between the horizontal force vector and the horizontal plane for the left leg, Ly = angle between the horizontal force vector and the sagittal plane for the left leg, abd = abduction, flex = flexion, L = left, R = right.

**Table 3:** Angle values (mean in degrees ± SD) in variables dependent on the level of amputation

Variable	Swing phase	Participant 1	Participant 2	Participant 3	Participant 4
X factor	takeaway	47 ± 1.2	47 ± 7.2	67 ± 1.2	35 ± 6.2

O factor	takeaway	11 ± 1.1	10 ± 1.1	7 ± 2.3	1 ± 0.4
	Early follow	−6 ± 1.4	−5 ± 1.3	−4 ± 1.4	4 ± 1.5
	Late follow	−15 ± 1.2	2 ± 1.3	−2 ± 1.2	5 ± 1.3

S factor	initial	−12 ± 1.4	−12 ± 1.2	−11 ± 1.1	−9 ± 1.1
	takeaway	35 ± 2.6	29 ± 3.6	30 ± 1.1	12 ± 2.0

Right knee flexion	initial	28 ± 1.2	19 ± 1.4	18 ± 1.2	5 ± 2.1
	takeaway	21 ± 2.0	7 ± 2.4	19 ± 1.3	5 ± 2.1
	Forward swing	19 ± 1.7	25 ± 1.2	18 ± 1.2	5 ± 1.1

Left knee flexion	Forward swing	22 ± 2.1	29 ± 2.1	18 ± 1.3	7 ± 2.2
	acceleration	16 ± 1.2	18 ± 1.4	13 ± 1.4	7 ± 2.4
	Early follow	15 ± 3.1	15 ± 1.4	13 ± 1.6	9 ± 2.3

Left elbow flexion	takeaway	61 ± 2.2	55 ± 4.1	65 ± 4.2	105 ± 3.1
	Forward swing	43 ± 3.5	42 ± 4.2	52 ± 4.1	68 ± 1.2
	Late follow	68 ± 1.3	55 ± 3.4	35 ± 3.1	108 ± 1.2

Right arm abd	acceleration	5 ± 1.4	6 ± 1.2	4 ± 1.3	6 ± 1.3

Left arm abd	initial	−12 ± 1.1	−5 ± 1.1	−9 ± 1.0	6 ± 1.3
	Forward swing	−20 ± 4.1	−7 ± 2.3	−3 ± 2.1	6 ± 1.8
	acceleration	−4 ± 1.4	−2 ± 1.2	−1 ± 1.1	16 ± 3.1

Left hip abd	Acceleration	79 ± 2.9	86 ± 2.7	82 ± 2.1	88 ± 1.1

Right hip abduction	Acceleration	85 ± 2.9	99 ± 3.7	98 ± 3.7	81 ± 1.1
	Early follow	82 ± 2.3	108 ± 3.4	95 ± 3.7	79 ± 1.2
	Late follow	96 ± 2.2	98 ± 2.6	87 ± 2.3	79 ± 2.1

Right hip flexion	Acceleration	23 ± 3.6	27 ± 3.4	10 ± 2.8	30 ± 4.5
	Early follow	12 ± 4.1	26 ± 4.6	6 ± 1.8	28 ± 3.6
	Late follow	0 ± 1.1	12 ± 2.1	−18 ± 1.5	25 ± 2.5

Left arm flexion	Forward swing	63 ± 2.1	52 ± 2.2	53 ± 3.1	45 ± 3.1
	Early follow	33 ± 2.5	22 ± 3.4	47 ± 2.6	94 ± 3.5

Force ratio (%)	Late follow	86 ± 9. 1	80 ± 7.1	78 ± 8.1	38 ± 9.2

Rx angle	forward swing	−81 ± 2.2	−80 ± 3.1	− 79 ± 2.4	84 ± 3.2

Lx	Early follow	77 ± 3.1	79 ± 3.2	80 ± 2.1	82 ± 2.2
	acceleration	85 ± 2.7	80 ± 2.5	81 ± 2.1	84 ± 2.2
	Late follow	87 ± 2.1	81 ± 2.3	80 ± 1.9	81 ± 1.2

Ly	early follow	85 ± 2.2	82 ± 2.6	87 ± 2.9	86 ± 1.1

SD = standard deviation, Rx = angle between the horizontal force vector and the horizontal plane for the right leg, Lx = angle between the horizontal force vector and the horizontal plane for the left leg, Ly = angle between the horizontal force vector and the sagittal plane for the left leg, abd = abduction.
